# Bayesian network model for identification of pathways by integrating protein interaction with genetic interaction data

**DOI:** 10.1186/s12918-017-0454-9

**Published:** 2017-09-21

**Authors:** Changhe Fu, Su Deng, Guangxu Jin, Xinxin Wang, Zu-Guo Yu

**Affiliations:** 1 School of Mathematics and Computational Science, Xiangtan University, Xiangtan, 411105 China; 2 School of Mathematics and System Science, Shenyang Normal University, Shenyang, 110034 China; 3 Center of Systems Biology and Bioinformatics, Wake Forest School of Medicine, Winston-Salem, NC 27157 USA

**Keywords:** Protein interaction, Genetic interaction, Biological pathway, Bayesian model

## Abstract

**Background:**

Molecular interaction data at proteomic and genetic levels provide physical and functional insights into a molecular biosystem and are helpful for the construction of pathway structures complementarily. Despite advances in inferring biological pathways using genetic interaction data, there still exists weakness in developed models, such as, activity pathway networks (APN), when integrating the data from proteomic and genetic levels. It is necessary to develop new methods to infer pathway structure by both of interaction data.

**Results:**

We utilized probabilistic graphical model to develop a new method that integrates genetic interaction and protein interaction data and infers exquisitely detailed pathway structure. We modeled the pathway network as Bayesian network and applied this model to infer pathways for the coherent subsets of the global genetic interaction profiles, and the available data set of endoplasmic reticulum genes. The protein interaction data were derived from the BioGRID database. Our method can accurately reconstruct known cellular pathway structures, including SWR complex, ER-Associated Degradation (ERAD) pathway, N-Glycan biosynthesis pathway, Elongator complex, Retromer complex, and Urmylation pathway. By comparing N-Glycan biosynthesis pathway and Urmylation pathway identified from our approach with that from APN, we found that our method is able to overcome its weakness (certain edges are inexplicable). According to underlying protein interaction network, we defined a simple scoring function that only adopts genetic interaction information to avoid the balance difficulty in the APN. Using the effective stochastic simulation algorithm, the performance of our proposed method is significantly high.

**Conclusion:**

We developed a new method based on Bayesian network to infer detailed pathway structures from interaction data at proteomic and genetic levels. The results indicate that the developed method performs better in predicting signaling pathways than previously described models.

## Background

A cellular biological system is controlled by the molecules at different levels, such as protein phosphorylation or genetic variations, and their interactions. Protein interaction (i.e., protein-protein interaction) refers to physical interconnection between two or more proteins that occur in a cell, by which protein components can carry out most of cellular molecular processes [1]. Genetic interaction refers to functional relationship between two genes, which can be measured by the difference between the phenotype levels of double gene mutations and the expected neutral level evaluated by the corresponding single mutant phenotype level [2, 3]. The publicly available data sets, such as Biological General Repository for Interaction Datasets (BioGRID, https://thebiogrid.org/), Saccharomyces Genome Database (SGD, http://www.yeastgenome.org/), Human Protein Reference Database (HPRD, http://www.hprd.org/), Search Tool for the Retrieval of Interacting Genes/Proteins (STRING: http://www.string-db.org/) and so on, collect thousands of proteins and a few genetic interactions from several of species.

Given a great deal of these interaction data collected, it is of challenges to elucidate biological meaning behind the data, especially to identify biological pathways underlying the data [4]. A few methods and tools have been developed to predict signaling pathways based on protein interaction networks [5–8]. Several different studies utilized various biological data to discover regulatory networks [9–12] and reconstruct metabolic networks [13–16]. There are other methods that uncover pathway networks by integrating protein-protein interaction data and gene expression data [17–19]. In genetic interaction studies, the most important method is cluster analysis, grouping genes by the similar genetic interaction profiles [20–22]. Some other studies focus on aggravating or alleviating relationships between related gene groups [23–25]. In order to automatically identify detailed pathway structures using high-throughput genetic interaction data, the activity pathway network (APN) was developed [26]. However, these available approaches cannot fully take advantages of the complementarity between protein and genetic interaction data to infer the biological pathway structures.

In this paper, we present a Bayesian model that integrates high-throughput protein and genetic interaction data to reconstruct detailed biological pathway structures. The model can organize related genes into the corresponding pathways, arrange the order of genes within each pathway, and decide the orientation of each interconnection. Based on protein interaction network, the model predicts detailed pathway structures by using genetic interaction information to delete redundancy edges and reorient the kept edges in the network. Similar to APN [26], our model represents a biological pathway network as a Bayesian network [27], in which each node presents the activity of a gene product. Different from APN that drew network sample from complete network, our method introducing protein interaction networks as underlying pathway structures. In addition, a scoring function is defined by gene pairwise score, which can avoid the unadjusted balance between gene pairwise score and edge score in the APN. Thus, our model is able to improve computational efficiency of stochastic simulation algorithm and overcome the limitation of APN that some edges in the results are difficult to interpret. In our model, each edge in the network can capture physical docking, and represent functional dependency.

## Methods

### Bayesian network

We model a pathway network as a Bayesian network that is a directed acyclic graph. The activity of a gene is assigned to a node in the network [26]. The edge in the network is an interaction in protein interaction network. Additionally, it presents the conditional dependency between the nodes connected as well. The experiments of genetic interaction are not for detection of the influence between pairwise genes but for measurement of impact of mutation of these two genes on phenotype of interest. Thus, it is impossible to evaluate conditional probability distribution between the nodes of the Bayesian network, and the standard Bayesian learning methods lost their efficacy. Here, we only utilize conditional independence assumptions of the Bayesian network theory to construct a network that can represent independence assumptions hidden in the gene interaction data. As in Ref. [26], based on the independence assumptions, it is elucidated that given the activity level of *X*, the fitness level is independent of the activity level of *Y*, if gene *X* is fully epistatic to gene *Y*. The constructed network can encode a linear pathway substructure between *X* and *Y*, in which *Y* must be the father node of *X*, that is, the direction of edge between is decided.

### Scoring

**Fig. 1:**
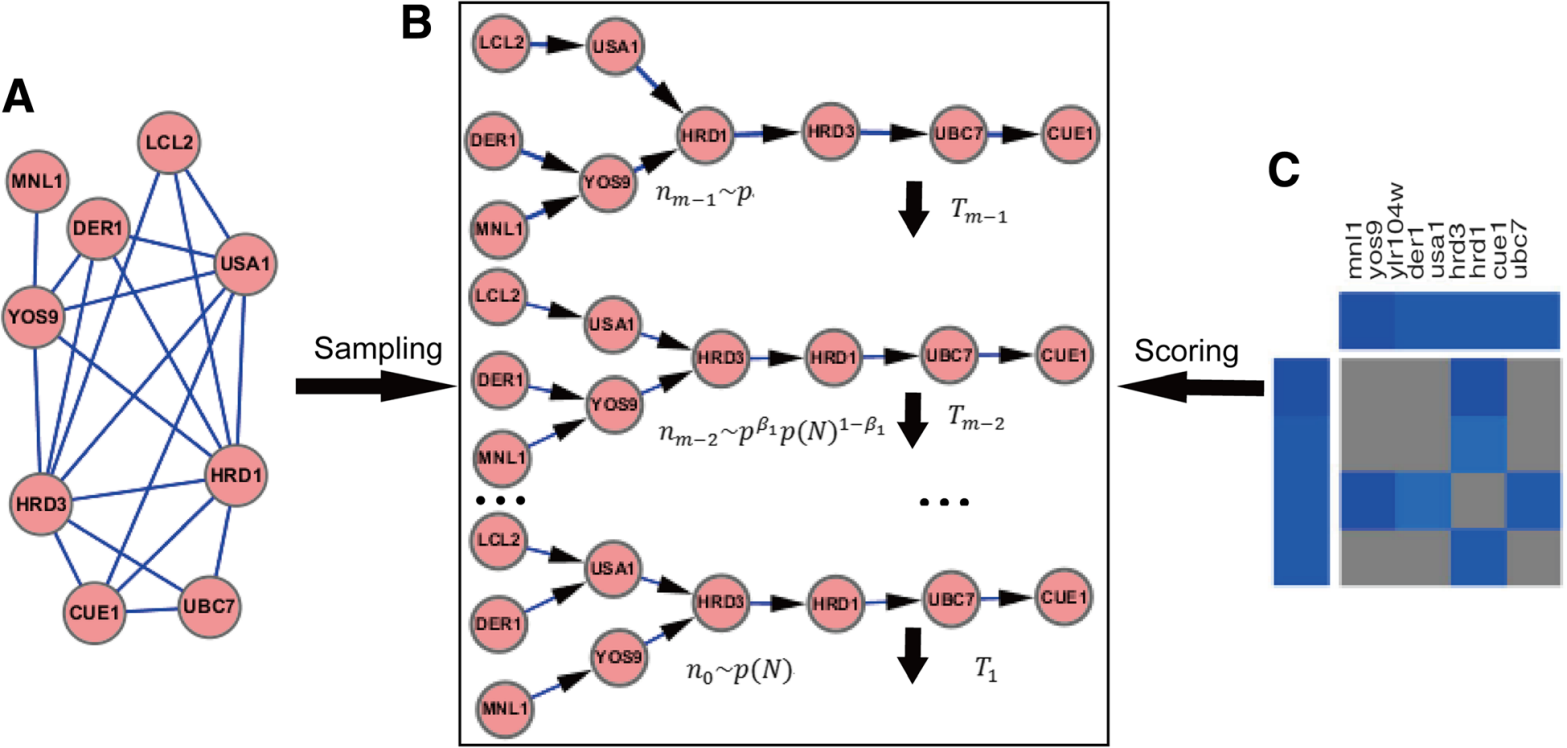
The brief procedure of the annealed importance sampling. (**a**) Protein interaction network of a gene set. It is not necessary that this network has to be a connected graph. (**b**) The procedure sampling *N*_*k*_ from *p(N)*. This annealed run generates a sequence of networks: *n*_*m* − 1_, *n*_*m* − 2_, …, *n*_0_, which are drawn from *p*_*j*_ kept by temperature *T*_*j*_ for the annealing run and genetic interaction score (*j* = 0,  … , *m* − 1). Where,${p}_j={p}^{1-{\beta}_j}p{(N)}^{\beta_j}$, 1 = *β*_0_ > *β*_1_ >  …  > *β*_*m* − 1_ = 0, the *p* is a uniform distribution from which an initial network *n*_*m* − 1_ can be drawn easily. Then let *N*_*k*_ = *n*_0_, and save the weight *ω*_*j*_. (**c**) Genetic interaction profiles. Using the profiles, score of candidate network can be computed, and the distribution *p(N)*, which is proportional *f(n)* shown as eq. (2), can be defined

**Fig. 2:**
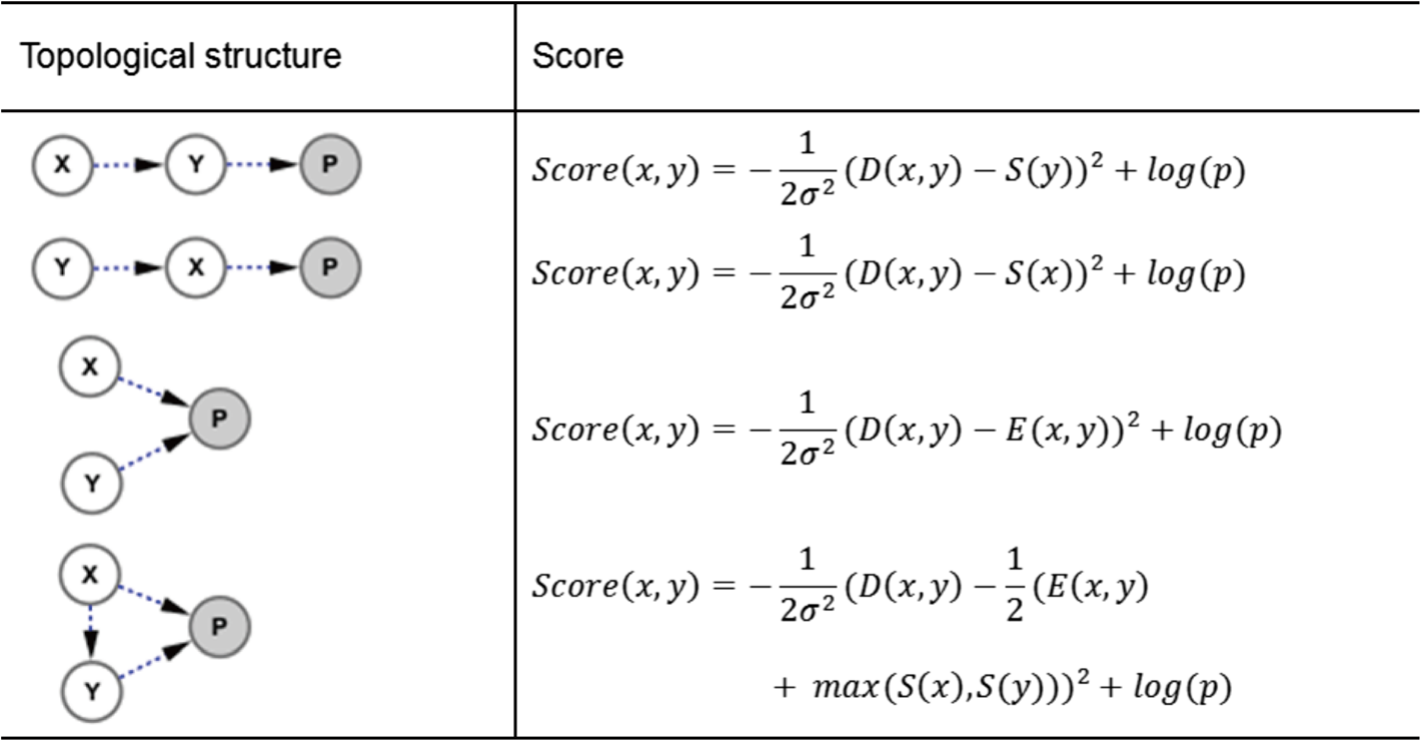
Topological structure and score of gene pair. For a pair of genes (*X*, *Y*), node *P* represents the measured quantitative phenotype. Dotted line means that the connection may be not a direct edge in the pathway network. Based on Gaussian distribution, the score for each topological structure is defined by the probability of actual double mutation measured phenotype value (fitness). Where, *D(x, y)* is (*x*, *y*) double mutant fitness, *S(.)* is single mutant fitness, and *E(x, y)* is typical genetic interaction value defined as [22]. The variance *σ* ^2^ is the empirical variance of repeated experiments, and *p* is the prior probability for each given topological structure

For a candidate pathway network (Fig. 1b) sampled from protein interaction network, we score it in term of genetic interaction quantitative measurement using method in Ref. [26]. For every pair of genes, there are four topological structures and their local scores shown in Fig. 2. Despite the larger score indicating the more possible local structure for each gene pair, we still need every one of four scores to find the optimal global structure. We computed the four possible scores for each pair of genes before all the steps to improve computation efficiency.

Using the scoring methods in Fig. 2 and dataset *D* of genetic interaction and protein interaction, we can compute a local score for every pair of genes in a candidate pathway network *N*, and sum up all of the scores for all pairs to define the global score function *f(N)*, to which the Bayesian network posterior probability distribution *p(N|D)* is proportional, shown as eq. (1). In Bayesian network theory [27], a network *N* with the higher posterior probability or global score should be more accord with the data set.

1 $$ f(N)=\exp \left(\sum_{x\ne y\  in\ N} Score\left(x,y\right)\right) $$

Different from study of Ref. [26], we do not include every edge score in *f(N)*, because the edge in our network represents protein interaction that insures its existence. Then, it avoids the dilemma how to adjust the balance between the two scores.

### Sampling

We utilized annealed importance sampling [26, 28] to learn the pathway structure by the above distribution *p* (*N*| *D*) ∝ *f*(*N*). The annealed importance sampling approach can assign weights to pathway networks sampled by simulated annealing schedules, then to evaluate that converge to the real network structure. The approach is appropriate for sampling *N* from multi-modal distributions *p* (*N*| *D*) or abbreviated to *p(N)*, since its independent sampling method can overcome some problems of convergence and autocorrelation in general Markov chain Monte Carlo (MCMC) samplers. Figure 1 presents the brief procedure of an annealing run of the annealed importance sampling.

### Pooling

After *K* annealing runs, the sampler generates *K* pathway networks and their weights. Then we can compute the confidence for any given substructure *s*, shown as

2 $$ C(s)=\frac{\sum_{k=1}^K{\omega}_kI\left(s\subset {N}_k\right)}{\sum_{k=1}^K{\omega}_k} $$

Where *I*(∙) is the indicator function, *N*_*k*_ is the sample at the *kth* annealing run, and *ω*_*k*_ is the important weight. Based on the theory of annealed importance sampling, we can compute confidences of all structure forms of an interesting gene subset, and choose the maximal one as the possible detailed pathway structure of the subset.

### Pseudo-code for pathway network reconstruction

**Input:** Matrix P: protein interaction network

- Vector S: signal mutation levels
- Matrix D: double mutation levels
- Matrix E: typical value for double mutation levels
- Vector T: temperatures for the annealing run
- Integer K: number of parallel annealing runs
- Some optional parameters

**Output:** Matrix of directed pathway networks and their weights

**Procedure:**

Compute all scores for every possible gene pair by inputs of genetic interaction data

Compute *p(N)* by scores of gene pairs in *N*

m = length(TV)

Design distributions *p*_*j*_(*j* = 0,  … , *m* − 1) (as Fig. 1) to approach *P(N)*

For *i = 1* to *K*:

- Sample initial network *n*_*m* − 1_ from uniform distribution *p*_*m* − 1_
- For *j = m-2* to *0*:
  - Generate *n*_*j*_ from *n*_*j* − 1_ by uniform distribution over P
  - Accept *n*_*j*_ according to Metropolis–Hastings algorithm by *T*_*j*_ and *p*_*j*_
  - Update importance weight
- Save network *N*_*i*_ and its weight *ω*_*i*_

Return networks *N*_*i*_ (*i* = 1,  … , *K*) and their importance weights

Specify interesting pathways and compute their confidence

The MATLAB codes of our algorithm can be freely downloaded at [29].

## Results and discussion

We applied our developed method to the genetic interaction measured by the protein folding in the endoplasmic reticulum [22] and the corresponding protein interaction network. The genetic interaction data set contains 444 queries crossed to the same 444 array strains from the budding yeast, *Saccharomyces cerevisiae*, and keeps available 86,396 raw double mutants from the 444 × 444 genetic interaction pairs [22]. Another genetic interaction data are from the coherent subsets of the global genetic interaction network [30], including 198 single mutants and 30,256 double mutants. We used regression method to predict the missing genetic interaction data from known genetic interaction profiles. The protein interaction data of the above gene set are downloaded from BioGRID till December 2016.

Due to the fact that the raw measurements of genetic interaction data are limited in publicly available databases, we applied our developed method to an available data set from Ref. [22]. Though there are some raw measurement data sets in Refs. [2, 30], either smaller number of samples or the higher sparsity makes it infeasible to apply our method to these data sets. That also explains why few available methods were designed to reconstruct pathways by integrating genetic interaction and protein interaction data. We compared our results with those predicted by the APN to validate the advantage of our method.

**Fig. 3:**
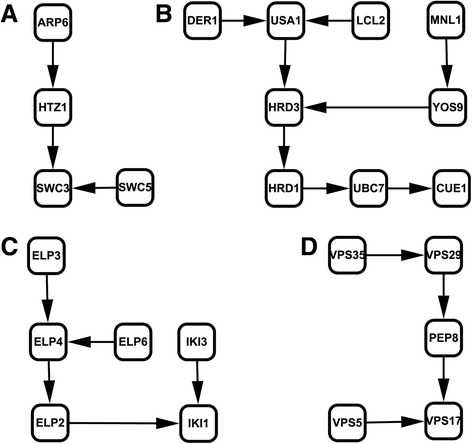
Substructures referred by our method. (**a**) Part of SWR complex. (**b**) ERAD pathway. Our method can place most of ERAD genes in appropriate position of the pathway. (**c**) Elongator complex. (**d**) Retromer complex

In our method, we modeled the pathway network as a Bayesian network. The sampling algorithm of annealed importance is applied to curate networks with the probability distribution defined by genetic interaction data, and simultaneously assign weights to them. And the corresponding protein interaction network of the genes in genetic interactions was used to represent underlying sample population, interpreting existence of potential edges in the sampled networks. Using these sampled networks and their assigned weights, we can estimate the detailed structure of the gene subset with high confidence (see Methods). Two substructures reconstructed by our method are shown in Fig. 3. Though the genetic interaction data for SWR complex are not complete, our approach still pools the existing genes together (Fig. 3a). It precisely reconstructs the known functional dependencies of ERAD pathway (Fig. 3b).

**Fig. 4:**
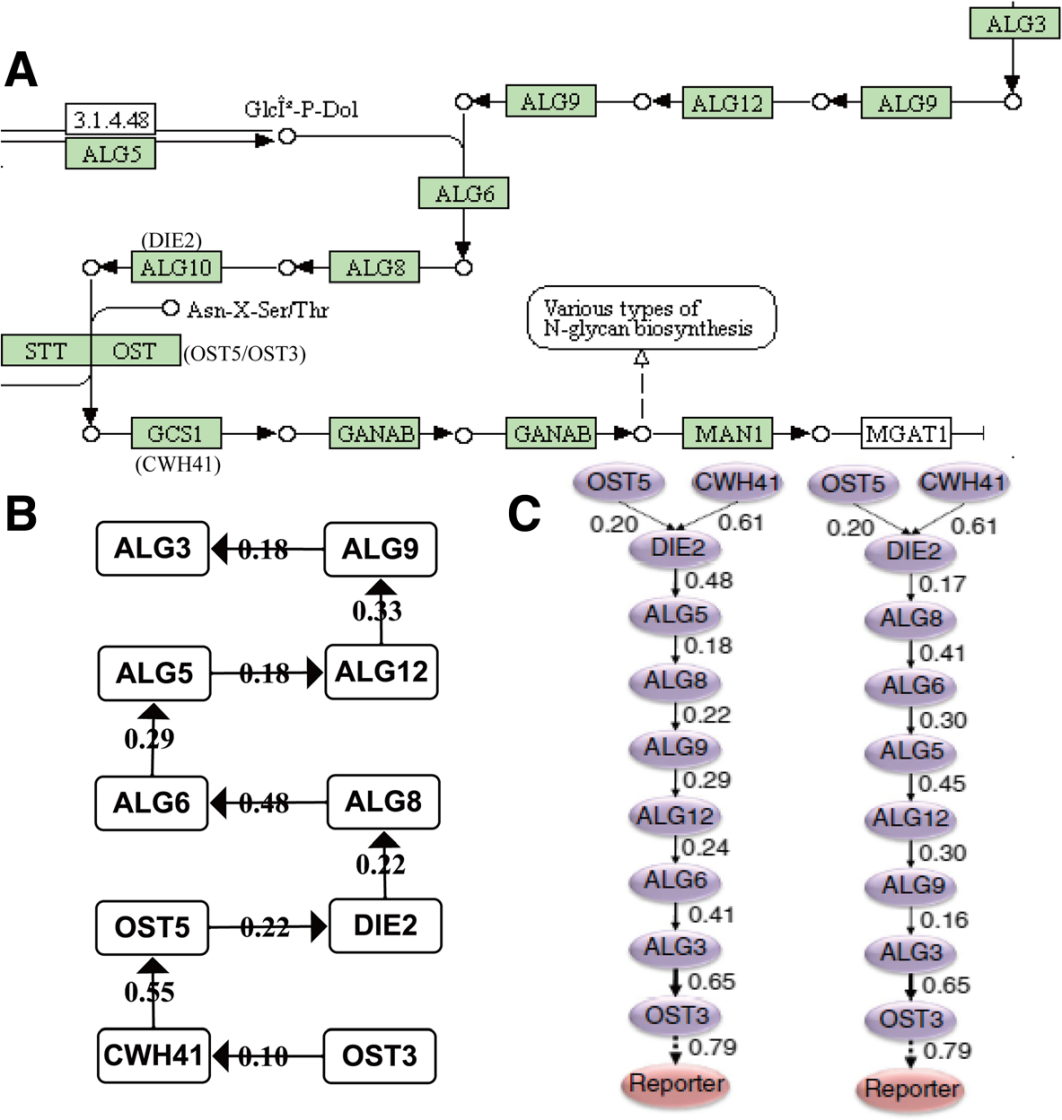
Comparison of N-Glycan biosynthesis pathways. The mechanisms of genetic interaction dependency can make the reverse ordering in (**b**) and (**c**). (**a**) The true ordering of N-Glycan biosynthesis pathway substructure from KEGG. (**b**) The linear structure of the pathway discovered by our model with high confidence. It can be found that DIE2 is not connected with CWH41 directly, because there is not the protein interaction in current database. And almost of all genes are on the correct place. (**c**) The structure from APN

We compared N-Glycan biosynthesis pathway substructure reconstructed by our model with the result of APN (Fig. 4). The detailed structures of the pathway from our model (Fig. 4b) and APN (Fig. 4c) [26] are very similar. Both of them are similar to the true one in Kyoto Encyclopedia of Genes and Genomes (KEGG, http://www.kegg.jp). One obvious difference is the place of OST3 that is incorrectly placed in APN (Fig. 4c). It may be due to the scoring function of APN based on edge score that strengthens the confidence of edge *(ALG3, OST3)*. The edge from ALG3 to OST3 has a high confidence, 0.65, indicating that APN really cannot interpret some edges in its result. Moreover, the orders of genes from our model and APN may be reverse to the true one [31] because the mechanisms of genetic interaction dependency are represented by phenotype (the unfolded protein response or fitness). Intriguingly, the OST3 position is correctly predicted in our method. It indicates the power of our developed method by integration of protein interaction data. However, we still found the limitation of the protein interaction data. The edge *(CWH41, DIE2)* is not presented in our result, because the corresponding protein interaction is not found in currently used protein interaction databases. In future, we are planning to include more predicted protein interaction data from STRING, and design parallel computing in high-performance computers to improve the performance.

**Fig. 5:**
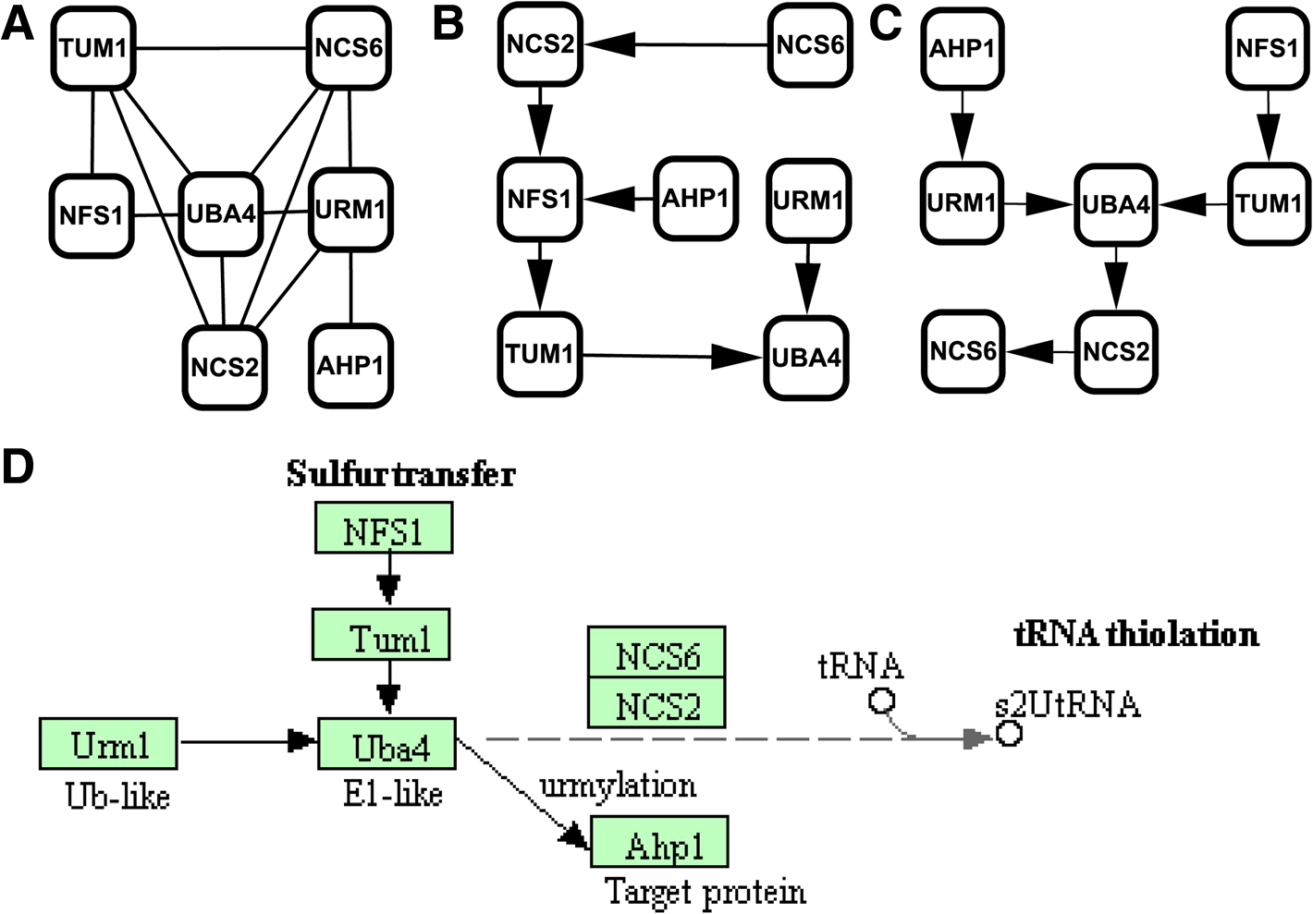
Comparison of Urmylation pathways. (**a**) The protein interaction network of the pathway from BioGRID. (**b**) The substructure of the pathway from APN. (**c**) The substructure of the pathway reconstructed by our model with high confidence. (**d**) The true ordering of the pathway substructure from KEGG

We also applied our model to infer pathways from another available data set of a global genetic interaction profiles [30]. From about 5.4 million gene pairs, we only selected coherent subsets in which the gene pairs have the high Pearson correlation coefficients, for our method based on annealed importance sampling is not suitable for so large data set. Using our model, we reconstructed three substructures, that is Urmylation pathway (Fig. 5c), Elongator complex (Fig. 3c), and Retromer complex (Fig. 3d). In Fig. 5, we compared our developed method with APN. The edge (NFS1, NCS2) presented in results of APN, as shown in Fig. 5b is difficult to interpret. However, our result in Fig. 5c is consistent with protein information from BioGrid as shown in Fig. 5a. The interactions of UBA4, NFS4, and NCS2 were predicted by our method. The edge (UBA4, AHP1) in Fig. 5d is not inferred by these two methods. For our model, the reason may be the incompleteness of protein interaction network that is the main weakness of our model.

## Conclusions

In this paper, we propose a Bayesian network model to identify pathway structures by integrating protein interaction with genetic interaction data. Our approach makes use of the complementarity between protein (physical) and genetic (functional) interaction data to refer the biological pathway structures. We define a scoring function by which the sampling algorithm of annealed importance can draw some pathway networks and their weights that are used to evaluate the candidate pathway structures. The results show that our model can predict the pathway structures more accurately.

## Abbreviations

APN: Activity pathway networks
BioGRID: Biological general repository for interaction datasets
ERAD: ER-Associated degradation
HPRD: Human protein reference database
KEGG: Kyoto encyclopedia of genes and genomes.
MCMC: Markov chain Monte Carlo
SGD: Saccharomyces genome database
STRING: Search tool for the retrieval of interacting genes/proteins
